# Geographical Patterns in Cyanobacteria Distribution: Climate Influence at Regional Scale

**DOI:** 10.3390/toxins6020509

**Published:** 2014-01-28

**Authors:** Frédéric Pitois, Isabelle Thoraval, Estelle Baurès, Olivier Thomas

**Affiliations:** 1 Limnologie sarl, 16 rue Paul Langevin, Rennes 35200, France; E-Mail: isabelle.thoraval@limnosphere.com; 2 Ecole des Hautes Etudes en Santé Publique de Rennes (EHESP), Sorbonne Paris Cité, Avenue du Professeur Léon Bernard- CS 74312, Rennes Cedex 35043, France; E-Mails: estelle.baures@ehesp.fr (E.B.); olivier.thomas@ehesp.fr (O.T.); 3 Institut National de la Santé Et de la Recherche Médical (INSERM), Unité 185, Institut de Recherche Santé Environnement et Travail (IRSET), Laboratoire d’Etude et Recherche en Environnement et Santé (LERES), Rennes 35043, France

**Keywords:** cyanobacteria, microcystin, toxins, climate change

## Abstract

Cyanobacteria are a component of public health hazards in freshwater environments because of their potential as toxin producers. Eutrophication has long been considered the main cause of cyanobacteria outbreak and proliferation, whereas many studies emphasized the effect of abiotic parameters (mainly temperature and light) on cell growth rate or toxin production. In view of the growing concerns of global change consequences on public health parameters, this study attempts to enlighten climate influence on cyanobacteria at regional scale in Brittany (NW France). The results show that homogeneous cyanobacteria groups are associated with climatic domains related to temperature, global radiation and pluviometry, whereas microcystins (MCs) occurrences are only correlated to local cyanobacteria species composition. As the regional climatic gradient amplitude is similar to the projected climate evolution on a 30-year timespan, a comparison between the present NW and SE situations was used to extrapolate the evolution of geographical cyanobacteria distribution in Brittany. Cyanobacteria composition should shift toward species associated with more frequent Microcystins occurrences along a NW/SE axis whereas lakes situated along a SW/NE axis should transition to species (mainly Nostocales) associated with lower MCs detection frequencies.

## 1. Introduction

Since their appearance in the public health debate, cyanobacteria and their toxins have gradually become a major concern for public health authorities [1,2]. 

While relations between cyanobacterial blooms and eutrophication are widely acknowledged [3], the influence of meteorological parameters is of growing concern in a changing climate context. Some recent review articles outline that in hotter environments, cyanobacteria could be advantaged compared to other planktonic taxa, and health issues associated with cyanobacterial toxin occurrences could thus become more pregnant [4,5,6,7,8,9].

However, if a changing climate can lead to predictable consequences on larger time and/or geographical scales, extrapolating these changes to the short term and on local scale is still complex [10]. It can be noted, for example, that local, long term studies related to single lakes can lead to different or diverging conclusions [11,12,13], whereas all studies acknowledge that climate change should lead to major modifications in phytoplankton populations [14,15,16], although counter-examples exist [17].

Common abiotic parameters such as light and temperature have long been shown to influence selection and growth rates of potentially toxic species [18,19,20,21,22,23] and toxin biosynthesis [24,25,26,27,28]. These results emphasize the possible consequences of a large scale environment warming up on health hazards related to cyanobacteria [6,7,16,23,29,30,31].

In this context, this paper aims at studying climate influence on cyanobacteria on a regional scale in Brittany (north-western France). Preliminary studies have already shown that the local oceanic-type climate of Brittany has been warming up for the last 30 years [32], and that cyanobacteria are widely encountered in most recreational lakes [33]. In the same time, available interannual monitoring data tend to show that cyanobacteria are increasingly present, with expansion parameters related to climate and lake morphology [34]. The present study is based on the observation that climate evolution at regional scale is of the same magnitude of latitudinal meteorological gradients, leading to the hypothesis that a comparison between eastern and western Brittany can give insights of the potential future situation regarding cyanobacteria (species composition, toxin occurrences) in a 30-year timespan.

## 2. Results and Discussion

### 2.1. Regional Climate Characteristics

**Figure 1:**
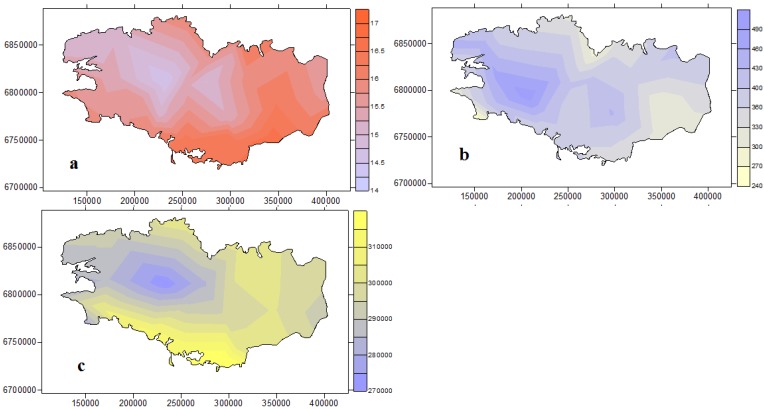
Meteorological parameters for the May–October period from 2004 to 2011. (**a**) Mean temperature (°C); (**b**) Cumulated pluviometry (mm); (**c**) Cumulated global radiation (kW/m²).

Brittany is characterized by an oceanic climate and mild conditions all year round, summarized in Table 1. These parameters follow a longitudinal gradient along a WNW-ESE axis, with a colder/wetter NW quadrant and drier/hotter SE quadrant (Figure 1). In the following study, only May–October conditions were considered for the evaluation of cyanobacteria distribution as no recreational water monitoring is conducted during the other months.

**Table 1:** Meteorological parameters expressed as monthly means from 2004 to 2011 in Brittany.

Parameter	December–March			May–October		
	Minimun	Mean	Maximun	Minimun	Mean	Maximun
Mean Temperature (°C)	3.7	6.6	9.2	12.2	15.9	20.1
Mean Pluviometry (mm)	49	84	119	25.5	61.1	137.3
Mean Global Radiation (kW/m²)	8.65	17.53	34.30	22.55	49.54	67.51

### 2.2. Regional Climate Projected Evolution

Interannual climate evolution was characterized for the 30-year timespan 1982–2011. Mean monthly data from the 34 meteorological stations nearest to the lakes (21 stations for global radiation) were approximated through linear regression curves. At regional scale, evolution rates for the May–October period show mean temperature and cumulated global radiation increases (+2.8 °C and +8 kW/m² for 100 year extrapolations); but no significant cumulated pluviometry evolution. These evolution rates are close to regional NW-SE climatic gradients (2 °C; 4 kW/m²; and 210 mm) and to interannual mean amplitudes (2.6 °C; 31 kW/m² and 154 mm) for the period 2004–2011. These results support the hypothesis that comparison between eastern and western Brittany can be a tool for evaluating the effect of climate change at regional scale in the short term.

**Figure 2:**
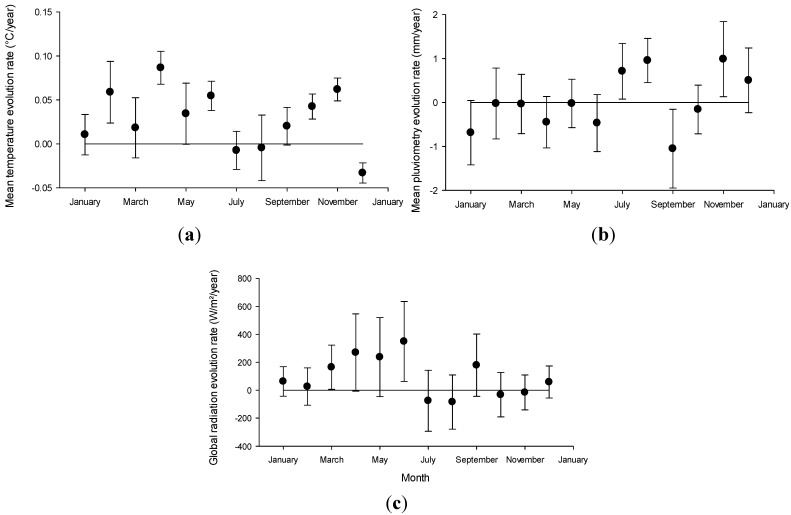
Climate parameters evolution rate extrapolated from 1982–2011 data (mean ± sd). (**a**) mean monthly temperature; (**b**) mean monthly cumulated rainfall; (**c**) mean monthly cumulated global radiation.

This regional evolution is associated with strong local variations: for a 30-year extrapolation, for example, site-specific evolution ranges from −0.6 to +2.3 °C, cumulated rainfall from −180 to +122 mm and cumulated global radiation from −4.7 to +11.5 kW/m². Faster evolution rates are concentrated in spring and early summer (Figure 2), with April to June showing the highest positive rates for temperature and global radiation, whereas in autumn months, only temperatures tend to increase. On the other hand, July and August, commonly the hottest months in oceanic climates, tend to show increasing rainfall and decreasing global radiation.

### 2.3. Cyanobacteria Distribution

**Figure 3:**
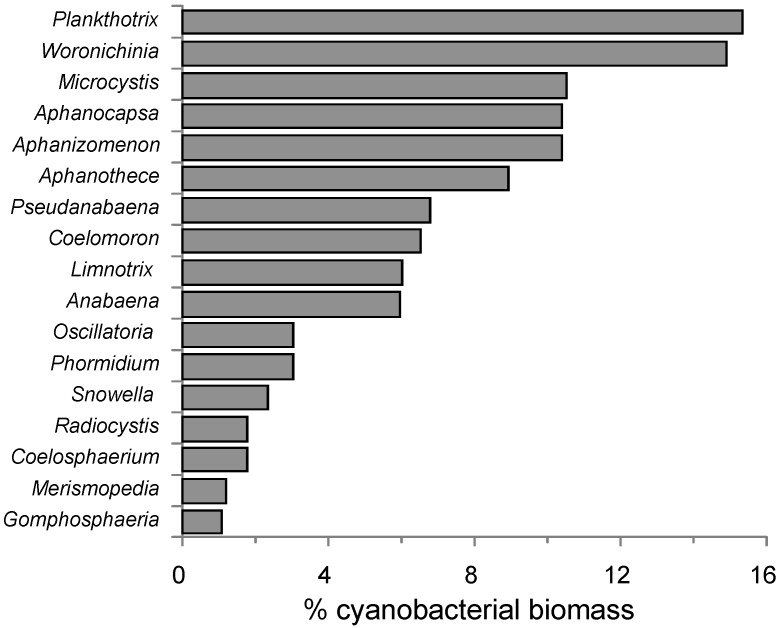
Dominant taxa expressed as % total cyanobacteria biovolume (taxa with % biovolume <1% omitted for clarity).

In the 2004–2011 data, 27 common genera accounted for 0.1%–16% of total cyanobacteria biovolume (Figure 3). The highest contribution could be credited to *Planktothrix* and *Woronichinia* (15.5%–16%), whereas *Microcystis*, *Aphanocapsa*, *Aphanothece* and *Aphanizomenon* ranged from 9% to 11%. The genus *Anabaena* only accounted for 6.3% of cyanobacteria biomass, but was included in this analysis because of its potential toxicity. These seven generic groups accounted for 76% of all cyanobacteria biovolumes. In all subsequent analyses, *Aphanocapsa* and *Aphanothece* data were pooled together as identification confusions were considered possible.

**Figure 4:**
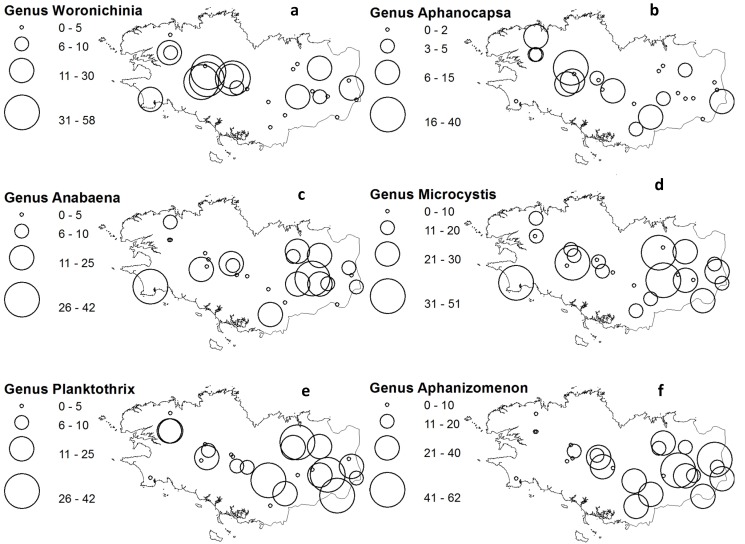
Geographical distribution of generic groups as % total cyanobacteria biovolume. (**a**) *Woronichinia*; (**b**) *Aphanocapsa* & *Aphanothece*; (**c**) *Microcystis*; (**d**) *Anabaena*; (**e**) *Planktothrix*; (**d**) *Aphanizomenon*.

Geographical distribution of these taxa was mapped with ESRI ArcView as local genus-specific mean % biomass for the period 2004–2011 (Figure 4). This illustrates regional tendencies, with higher % biomass for *Woronichinia* and *Aphanocapsa* in the north-western quadrant, for *Microcystis* and *Anabaena* from centre to east, whereas *Planktothrix* and *Aphanizomenon* are higher contributors in the south-east quadrant.

### 2.4. Microcystin Occurrences

**Figure 5:**
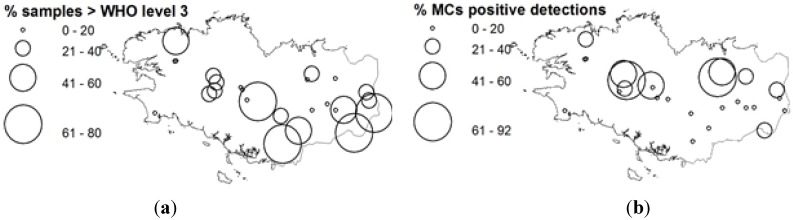
(**a**) WHO level 3 occurrence frequencies (% samples > 100,000 cell/mL); (**b**) Microcystin (MC) detection frequencies (% samples with MCs > MQL).

Microcystins (MCs) detection frequencies, once mapped, appeared concentrated in the centre-north part of Brittany, *i.e.*, outside of the SE quadrant concentrating the highest % biomass of most known MC-producing taxa. At the same time, MC detection frequencies could not be related to common public-health parameters such as WHO alert level 3 observation frequencies (*i.e*., cell density > 100,000 cell/mL: r² = −0.45), total cyanobacteria cell densities (r² = −0.01), known MC producers cell densities (r² = −0.04), *etc.* (Figure 5).

### 2.5. Cyanobacteria-Climate Relations

**Figure 6:**
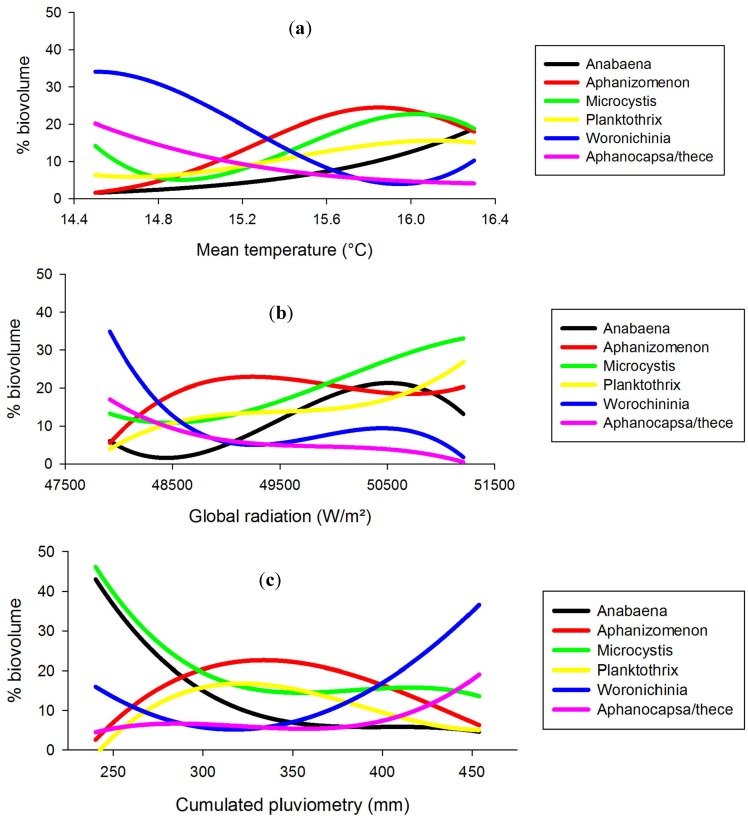
Generic % biovolume distribution *vs.* (**a**) Mean temperature; (**b**) Cumulated Global Radiation; (**c**) Cumulated pluviometry (data points omitted for clarity).

Contribution to biomass for each generic group was examined in relation to meteorological parameters in order to determine dependencies toward regional climate gradients. All relations are summarized in Figure 6 as second order polynomial curves obtained with SPSS SigmaPlot 12.

Three dominant areas could be distinguished: a colder, wetter, less sunny area dominated by *Aphanothece/Aphanocapsa* and *Woronichinia*, a hotter, drier, sunny area dominated by *Microcystis* and *Anabaena*, and an intermediate, transitional area associated with *Aphanizomenon* and *Planktothrix*. This distribution is in accordance with cyanobacterial growth rate and thermal optima as exposed in [35,36].

Climatic optima, summarized in Table 2, were extrapolated for each genus and defined as the parametric value leading to the highest % biovolume. Some of these optima can be deducted to be out of our data range when no inflexion could be observed on the distribution curve.

### 2.6. Microcystin-Climate Relations

**Figure 7:**
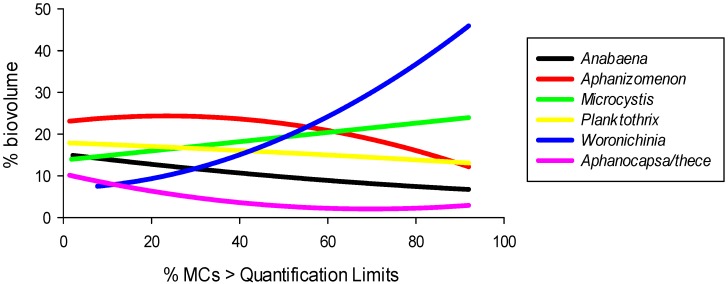
MC detection frequencies according to generic composition (as % total biomass).

Microcystins (MCs) detection frequencies could not be directly related to climatic parameters (*vs*. temperature: r² = −0.28; *vs.* rainfall r² = 0.43; *vs.* global radiation: r² = −0.01), but a relation may be highlighted between MCs and generic groups, leading to an indirect relation with climatic areas (Figure 7). This can be explained by the ability of each generic group to host potential toxin producers in its own distribution area.

MC detections were negatively related to % biovolume for *Anabaena*, *Aphanizomenon* and *Planktothrix*, and positively correlated with *Woronichinia* and *Microcystis*. Accordingly, positive detection frequencies reached 50% of analysed samples on one hand if *Microcystis* and/or *Woronichinia* accounted for at least 20% of total cyanobacteria biovolume, and on the other hand if *Anabaena* accounted for less than 10% biovolume, and *Aphanizomenon* or *Planktothrix* accounted for less than 17%–20% biovolume.

**Table 2:** Taxa distribution criteria extrapolated from May–October climatic data.

Genus	MT	CGR	CP
	°C	kW/m²	mm
Aphanothece/capsa	<14	<48	>455
Woronichinia	14.4	<48	>455
Aphanizomenon	15.8	49.2	335
Planktothrix	16.1	>51.2	320
Microcystis	16.1	>51.2	<240
Anabaena	>16.5	50.5	<240

Notes: MT: mean temperature; CGR: cumulated global radiation; CP: cumulated pluviometry.

These results were obtained from regional-scale, interannual data, and should not be interpreted as an affirmation that MCs are only produced by *Microcystis* and *Woronichinia* in Brittany. Moreover, no analysis was used to verify which taxa were observed in samples showing positive MC detection. These results are indicative of higher MCs occurrences in two nearly opposite climatic domains, *i.e.*, the colder/wetter *Woronichinia* area *vs.* the drier/hotter *Microcystis* area.

### 2.7. Cyanobacteria and Microcystin Domain Projections

Projected data from this 30-year extrapolation were inserted in a simple multiple linear regression model for all 26 lakes. This model was intended to evaluate the evolution of *Microcystis* and *Woronichinia* domains, in order to estimate the possible modifications of positive MC detection frequencies. The results show a main dependency of *Woronichinia* (r² = 0.74, p = 0.02) to pluviometry (p = 0.02), whereas *Microcystis* (r² = 0.42, p = 0.05) are mainly explained by global radiation (p = 0.04). Higher *Microcystis* contribution to biovolume tend however to be underestimated.

Despite this limitation, extrapolated biovolumes for a 30-year projection show that *Microcystis* domain could expand from eight to 20 out of 26 lakes, whereas *Woronichinia* domain should decrease from seven to three lakes. Intermediate (*Aphanizomenon*/*Planktothrix*) domains would then decrease from eleven to three lakes: nine intermediate domain lakes are projected to join *Microcystis*/*Anabaena* domains, whereas four *Woronichinia/Aphanothece/Aphanocapsa* lakes are projected to transfer to *Aphanizomenon/Planktothrix* conditions. This could lead to an extension of frequent MC detections (>50% samples) from 15 to 23 lakes, *i.e.*, nearly a 50% increase. The studied lakes are mostly situated along a WNW-ESE axis. When our multiple linear regression model is used at full regional scale (*i.e*., integrating climatic parameters from the NE and SW quadrants), the same 30-year extrapolation shows that the intermediate domain associated with *Aphanizomenon/Planktothrix* is projected to expand in replacement of the current *Woronichinia* domain, whereas most current intermediate or *Microcystis/Anabaena* lakes should remain in the same category. This result however could not be verified, as insufficient continuous monitoring data were available for these two quadrants.

These results tend to show that climate change at the current observed rate is expected to involve changes in cyanobacteria distribution patterns at regional scale. These modifications can appear to be complex at local scale, especially with the observed decrease of July and August temperature and global radiation, but the extrapolated regional evolution seems in broad agreement with observations collected from site-specific studies around the world [6,15,16]. From another point of view, the projected conservation of the hottest/driest *Microcystis* domain in oceanic climate can explain why no significant evolution in cyanobacteria occurrences or species composition could be highlighted in the 14-year study of Agueira Reservoir [17].

From 2004 to 2011, no nutrient monitoring was conducted in these lakes. As a consequence, no relevant trophic status data could be integrated to this work, and our conclusions are only based on climatic data. Other parameters can influence cyanobacteria distribution, such as lake morphology and/or nutrient form availability to phytoplankton [37,38]. Nutrient data should be included as a priority in any cyanobacteria monitoring program.

## 3. Experimental Section

**Figure 8:**
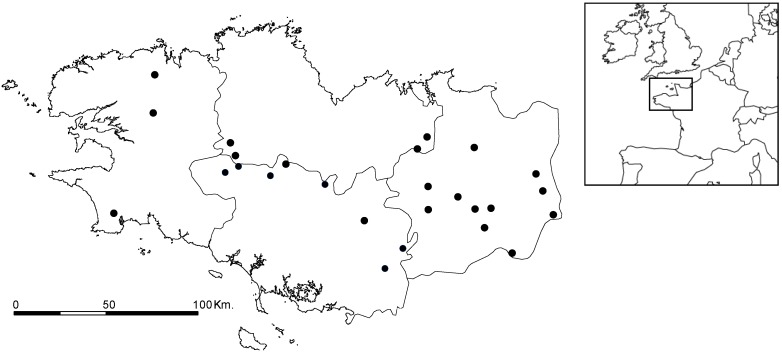
Localization of the 26 lakes studied in Brittany (western France).

This study is based on public health weekly survey data from recreational lakes monitored every year from 2004 to 2011 in Brittany, north-western France (Figure 8). These sites were selected according to cyanobacteria survey continuity criteria: 26 sites out of a total of 40 lakes monitored for cyanobacteria from 2004 to 2011 could be considered as continuously monitored from June to September, *i.e.*, with at least seven years of monitoring out of eight, with a sampling frequency of at least every two weeks. The lakes characteristics range from 0.2 to 51 × 10^6^ m^3^, with depths from 2 to 45 m and watershed areas from 0.2 to 676 km².

Monthly meteorological data (precipitation rates, temperature, global radiation intensity, *etc.*) were collected from Météo France database (Climathèque) from 1982 to 2011. Meteorological data were gridded and mapped with Golden Software Surfer 8 and ESRI ArcGis Desktop 9, whereas regional climate evolution rates were estimated with SPSS Sigma Plot 12.

Only four out of 26 lakes were subject to nutrient monitoring prior to this study. As a consequence, no relevant trophic status data could be integrated to this work.

Cyanobacteria data, *i.e.*, cell densities, species composition and microcystin concentrations, were collected from the regional public health authorities (French Agence Régionale de Santé (ARS) of Brittany). All samples were analyzed by four different local laboratories for cyanobacteria composition and microcystin concentration.

Cyanobacteria were identified according to [39,40,41] and counted with light microscope according to [42]. All cell densities data were first converted to biovolume with the use of mean cell dimensions and relevant geometric formulas [43], to account for differences in contribution from larger species associated with lower cell densities (e.g., *Microcystis*, *Woronichinia*, *etc.*) and smaller species associated with larger cell densities (e.g., *Aphanocapsa*, *Aphanothece*, *etc.*).

As biomass is a consequence of nutrient availability [3], all biovolumes were converted to percentage (cyanobacteria) biovolume in order to reduce any inter-site bias related to different trophic status. Generic biovolume contributions were mapped with ESRI ArcGis Desktop.

Microcystins (MCs) were analyzed either with LC/MS, HPLC or ELISA immunoassays depending of the laboratory. All toxin analyses involved a preliminary cyanobacteria cell lysis step with methanol [44] to account for total (dissolved and intra-cell) toxin concentrations. These methods quantification limits range from 0.05 to 0.2 µg/L. As no interlaboratory calibration data was available, all quantitative results were sorted as lower than (MCs < 0.2 µg/L) or higher than (MCs > 0.2 µg/L) method quantification limit, and then analyzed as detection frequencies for every lake.

## 4. Conclusions

Data analysis from eight years of monitoring 26 lakes shows that, at a regional scale, cyanobacteria tend to be associated with homogeneous geographical and climatic areas. In an oceanic climate context, such as Brittany, three main domains characterized by their dominant genera could be distinguished: a colder/wetter domain dominated by *Woronichinia/Aphanocapsa/Aphanothece*, a hotter/drier domain associated with *Microcystis/Anabaena*, and an intermediate domain combining *Aphanizomenon/Planktothrix* species.

These three domains appear to be separated by differences of 1 °C (mean temperature), 15 kW/m² (global radiation) and 105 mm (cumulated pluviometry). The similarity between the three cyanobacteria domains separation and regional or interannual meteorological amplitude can explain why lakes in seemingly geographical proximity can show discrepancies in their reaction to overall similar regional summer conditions, and why lakes in some parts of Brittany tend to host similar cyanobacteria populations every year. These lakes tend to share the extremes of regional gradient, and in this case are situated in *Woronichinia* or *Microcystis* domains.

Our results show that microcystin positive detections are associated with area dominated (as % biovolume) by larger Chroococcales (*Woronichinia* and *Microcystis*) whereas areas dominated by other potentially toxic genera (*Anabaena*, *Aphanizomenon* and *Planktothrix*) are negatively correlated with detection frequencies. A 30-year timespan extrapolation tends to show that *Woronichinia* domains should decrease whereas *Microcystis* domains should expand, leading to higher MCs detection frequencies in most of the 26 lakes of this study.

At regional scale, MC detections are projected to increase in the lakes following an eastward/southward axis, whereas lakes situated along a SW/NE axis could transition to an intermediate domain associated with lower MC detection frequencies. However, lower MC observations should not be extrapolated to other toxin families. The most common species encountered in this intermediate domain are *Aphanizomenon flos-aquae*, known as a potential producer of PSP, Anatoxin-A and Cylindrospermopsin [45,46,47,48,49]; *Aphanizomenon issatschenkoï*, a PSP and Anatoxin-A producer [50,51,52], and *Aphanizomenon gracile*, a PSP producer [52,53,54].

Only MCs are mandatorily monitored in recreational lakes in France, so no data for other toxins could be integrated in this study. Their synthesis however has already been reported to be affected by abiotic parameters such as light and temperature [24,25,26,27,28], and as a consequence it seems important to extend toxin monitoring to PSP, Anatoxin-A and Cylindrospermopsins in order to build relevant health risk management policies in a changing climate context.
